# Association Between the Expression of MicroRNA-125b and Survival in Patients With Acute Coronary Syndrome and Coronary Multivessel Disease

**DOI:** 10.3389/fcvm.2022.948006

**Published:** 2022-07-08

**Authors:** Gloria M. Gager, Ceren Eyileten, Marek Postula, Aleksandra Gasecka, Joanna Jarosz-Popek, Georg Gelbenegger, Bernd Jilma, Irene Lang, Jolanta Siller-Matula

**Affiliations:** ^1^Division of Cardiology, Department of Internal Medicine II, Medical University of Vienna, Vienna, Austria; ^2^Department of Clinical Pharmacology, Medical University of Vienna, Vienna, Austria; ^3^Department of Experimental and Clinical Pharmacology, Centre for Preclinical Research and Technology (CEPT), Medical University of Warsaw, Warsaw, Poland; ^4^Genomics Core Facility, Center of New Technologies (CeNT), University of Warsaw, Warsaw, Poland; ^5^1st Chair and Department of Cardiology, Medical University of Warsaw, Warsaw, Poland; ^6^Doctoral School, Medical University of Warsaw, Warsaw, Poland

**Keywords:** acute coronary syndrome, multivessel disease, microRNA, miR-125a, miR-125b, miR-223, long-term all-cause mortality

## Abstract

**Background:**

MicroRNAs (miRNA, miR) have an undeniable physiological and pathophysiological significance and act as promising novel biomarkers. The aim of the study was to investigate blood-derived miRNAs and their association with long-term all-cause mortality in patients with multivessel disease (MVD) suffering from acute coronary syndrome (ACS).

**Materials and Methods:**

This study was an observational prospective study, which included 90 patients with MVD and ACS. Expression of miR-125a, miR-125b, and miR-223 was analysed by polymerase chain reaction (PCR). Patients were followed-up for a median of 7.5 years. All-cause mortality was considered as the primary endpoint. Adjusted Cox-regression analysis was performed for prediction of events.

**Results:**

Elevated expression of miR-125b (>4.6) at the time-point of ACS was associated with increased long-term all-cause mortality (adjusted [adj.] hazard ratio [HR] = 11.26, 95% confidence interval [95% CI]: 1.15–110.38; *p* = 0.038). The receiver operating characteristic (ROC) analysis showed a satisfactory c-statistics for miR-125b for the prediction of long-term all-cause mortality (area under the curve [AUC] = 0.76, 95% CI: 0.61–0.91; *p* = 0.034; the negative predictive value of 98%). Kaplan–Meier time to event analysis confirmed an early separation of the survival curves between patients with high vs low expression of miR-125b (*p* = 0.003). An increased expression of miR-125a and miR-223 was found in patients with non-ST-segment elevation ACS (NSTE-ACS) as compared to those with ST-segment elevation myocardial infarction (STEMI) (*p* = 0.043 and *p* = 0.049, respectively) with no difference in the expression of miR-125b between the type of ACS.

**Conclusion:**

In this hypothesis generating study, lower values of miR-125b were related to improved long-term survival in patients with ACS and MVD. Larger studies are needed to investigate whether miR-125b can be used as a suitable predictor for long-term all-cause mortality.

## Introduction

MicroRNAs (miRNA, miR) are progressively evolving as an intriguing area of research and may have a huge potential as emerging biomarkers in clinical practice in the future (1, 2). MiRNAs constitute various small, non-coding RNA molecules with an average size of 22 nucleotides, which are responsible for the regulation of post-transcriptional silencing of target genes (3–7). However, Very recently, additional functions of miRNAs were proposed, such as transcriptional regulation (8) or even influencing protein functions (9, 10). Importantly, they play a crucial role in normal development of healthy subjects and dysregulation of their expression has been associated with a variety of disorders (1, 11, 12). The vast majority of miRNAs are intracellular, however, a substantial number has also been detected extracellularly. A wide range of miRNAs have become interesting–as miR-125a, miR-125b, and miR-223, for which a role in cardiac disorders, like acute coronary syndrome (ACS) has been shown (13–16).

Acute coronary syndrome is a collective term for three different conditions, comprising of ST-segment elevation myocardial infarction (STEMI), non-ST-segment myocardial infarction (NSTEMI) and unstable angina (UA) (17). The last two in turn can be combined under the expression non-ST-segment acute coronary syndrome (NSTE-ACS) (18). Over time, increased incidences of NSTEMI have been reported as opposed to the diagnosis of STEMI (19). The main distinction between both types has its origin in different pathophysiological processes. While a STEMI arises from a total vessel occlusion, NSTE-ACS is a consequence of a non-occlusive thrombus. Depending on the ACS subtype, treatment approaches vary. However, percutaneous coronary intervention (PCI) constitutes the gold standard therapy for ACS (18). In that setting, about 50% of the patients are concomitantly diagnosed with multivessel disease (MVD) (20), which is defined as the presence of a ≥50% stenosis in at least two major coronary arteries, detected by angiographic assessment. Simultaneous occurrence of ACS and MVD carries a poorer prognosis and treatment strategies are less well-established as compared to culprit-only ACS (21). Since there is evidence that chronic inflammation is associated with MVD (22), we focused on the sickest patient collective. Therefore, this study comprises only of patients, who were referred for PCI due to ACS, were diagnosed with MVD and received at least one drug-eluting stent (DES).

In the following study, we particularly focused on miR-125a, miR-125b, and miR-223, since previous research already reported on an involvement of miR-125b in cardiovascular pathologies (23–25), similar to miR-125a (26). In line, also miR-223 has been associated with a variety of cardiovascular disorders (27, 28). Further, we already performed a variety of bioinformatical analyses, where different roles regarding different miRNAs were investigated (5–7, 29–31). In addition, we have already found significant modulation of both miR-223 and miR-125 in patients with high vs. low on-treatment platelet reactivity after acute myocardial infarction in our previous study (32). The aim of the present study was to investigate, whether miR-125a, miR-125b, or miR-223 bear a prognostic value for prediction of long-term all-cause mortality and to analyze if the respective miRNAs show any differences related to the ACS subtype in patients with MVD. Due to the aforementioned relationship of inflammation with MVD, we further wanted to elaborate any association between the respective miRNAs and the most prevalently used marker for inflammation–C-reactive protein (CRP).

## Materials and Methods

### Study Design

The study was a prospective observational study, which included consecutive patients, who were diagnosed with ACS. The study took place at the Medical University of Vienna between 2012 and 2020. Whereas the active recruitment phase of the trial began in July 2012 and lasted until mid-June 2015, patients were followed-up until January 2020. The study protocol acts in accordance with the Declaration of Helsinki and was approved by the Ethics Committee of the Medical University of Vienna (approval number: 1051/2012). Expression of miR-125a, miR-125b, and miR-223 was measured in those 90 patients of the study cohort, who were diagnosed with MVD (26%). In summary, the present study investigated long-term outcome in consecutive ACS patients who underwent PCI. All patients had to present with an ACS at hospitalization, had to be at least 18 years of age, be able to provide of a written informed consent before study entry and had to receive a planned treatment with the potent P2Y_12_ inhibitor ticagrelor or prasugrel. Importantly, as platelets represent the primary source of circulating miRNA (5, 29–31), only patients on potent platelet inhibition were included to prevent bias. Exclusion criteria included the participation in interventional trials, refusal to provide of a written informed consent and an age of less than 18 years. Long-term survival data was obtained through queries of the Austrian death registry until January 2020.

### Study Endpoints

The primary endpoint was long-term all-cause mortality. Major adverse cardiac events (MACE) within the first year after discharge and long-term cardiovascular death were considered as our secondary endpoints. In this context, MACE was defined as non-fatal myocardial infarction, non-fatal stroke and cardiovascular death. Determination of the composite endpoint followed the current universal criteria (33, 34). Thrombolysis for Acute Myocardial Infarction (TIMI; minimal/minor/major) bleeding was considered as the safety endpoint. Further, we aimed to investigate an association between miR-125a, miR-125b, miR-223, and CRP.

### RNA Preparation and Detection and Quantification of miRNAs by Applying Quantitative Polymerase Chain Reaction

Extraction of blood plasma RNA was conducted through the mirVana PARIS Kit and subsequently diluted in a 1:10 ratio. The next step comprised of reverse transcribing 5 μL diluted RNA by using the TaqMan miRNA Reverse Transcription kit (ABI) in accordance to the instructions of the manufacturer. To detect expression of miRNAs by polymerase chain reaction (PCR), 3 μL of the dilution was used. TaqMan miRNA Assay kits (ABI) were applied for the corresponding miRNAs on a CFX384 Touch Real-Time PCR Detection System (BioRad Inc., Hercules, CA, United States). Subsequently, cel-miR-39 was added as a spike-in control. All reactions were performed in triplicate. The mean value was then calculated for all analyses to compensate for the variability of the methodology (35–37).

### Statistical Analysis

All data is presented as mean ± standard deviation (SD), median and interquartile range (IQR; range from the 25th to the 75th percentile), 95% confidence intervals (95% CI), numbers (n) and percentages (%) as applicable. All miR-125a, miR-125b, miR-223 values were converted to the decadic logarithm (log10) to normalize skewed data. Due to the explorative nature of the study, a prior evaluation of the statistical power has not been performed. Mann-Whitney *U*-test and the X^2^-test were used for comparison between groups. Receiver operating characteristic (ROC) analysis was performed for determination of miR-125b’s ability to predict long-term all-cause mortality. For those calculations, we followed the classical ROC analysis approach, however, alternative methods are also available as described previously (38). Regarding survival analyses, Kaplan–Meier curves and Mantel-Cox regression test were applied. To establish independent parameters influencing long-term all-cause mortality we used a multivariate Cox-regression analysis. Due to the limited number of events, only three parameters were established into the equation to prevent an overfitting of the model. In this context, the following variables were included: miR-125b > 4.6 (based on the ROC curve analysis), age ≥65 years and diabetes mellitus, as the latter two present the most powerful risk factors for developing MVD (39). For calculation of bivariate correlations between metric variables we made use of Spearman’s rho. All statistical analyses were operated using commercially available statistical software (IBM SPSS Statistics 25, IBM, Armonk, NY, United States).

### Group Stratification

To investigate the ability of miR-125b for prediction of long-term all-cause mortality, ROC analysis was applied. The cut-off point of miR-125b was defined by calculating the greatest sum of sensitivity and specificity of the ROC-coordinate points (40). Two groups were then stratified based on the cut-off value of 4.6–low miR-125b (≤4.6) and high miR-125b (>4.6). Likewise, the optimal cut-off points for miR-125a and miR-223 were calculated (5.0 and 7.4, respectively).

## Results

### Patient Demographics

Detailed baseline characteristics, including risk factors, past medical history, laboratory data, concomitant medication and ACS data are summarized in Table 1. Overall, majority of the patients (86%) were male and had a mean age of 61 years. Further, did 70% suffer from arterial hypertension and 57% from dyslipidemia, whereas diabetes mellitus was less common with 33%. Notable, 76% of the participants reported on smoking. Most patients (71%) were hospitalized due to STEMI, contrarily, 29% were diagnosed with a NSTE-ACS. At discharge, 100% were treated with aspirin. Further, the most common administered P2Y_12_ inhibitor was prasugrel (51%), followed by ticagrelor with 42%. Only 2% received clopidogrel (after switch from initial treatment with ticagrelor/prasugrel). Other frequently applied drugs included ß-blockers (88%), angiotensin-converting enzyme (ACE) inhibitors/angiotensin II receptor blockers (ARBs) (87%) and statins (93%).

**TABLE 1:** Patient demographics.

Patient demographics (*n* and %)	Overall *n* = 90 (100)	miR-125b ≤ 4.6 *n* = 63 (70)	miR-125b > 4.6 *n* = 27 (30)	*P*-value
miR-125b	4.1 ± 1.0	3.6 ± 0.7	5.7 ± 1.0	**<0.001**
Age (years) Sex (male), *n* (%)	60.9 ± 11.1 77 (86)	60.2 ± 10.5 57 (91)	62.5 ± 12.6 20 (74)	0.395 **0.043**
**Risk factors/past medical history *n* (%)**				
Body mass index	27.4 ± 5.5	27.1 ± 4.5	28.1 ± 7.4	0.090
Arterial hypertension	63 (70)	43 (68)	20 (77)	0.413
Dyslipidemia	51 (57)	36 (57)	15 (58)	0.962
Diabetes mellitus	30 (33)	23 (37)	7 (27)	0.384
Peripheral artery disease	4 (4)	2 (3)	2 (8)	0.350
Cerebrovascular disease	5 (6)	4 (6)	1 (4)	0.641
Chronic obstructive pulmonary disease	4 (4)	4 (6)	0 (0)	0.180
Smoking	68 (76)	50 (79)	18 (69)	0.306
Family history of CAD	40 (44)	30 (48)	10 (39)	0.430
Prior myocardial infarction	26 (29)	20 (32)	6 (23)	0.413
Prior PCI	17 (19)	14 (23)	3 (12)	0.231
**Laboratory data (mean ± SD)**				
White blood cell count (x10^9^/L)	10.6 ± 3.6	10.6 ± 3.6	10.8 ± 3.5	0.761
Platelets (x10^9^/L)	230.9 ± 57.3	228.1 ± 56.1	237.4 ± 60.6	0.657
Hemoglobin (g/dL)	14.2 ± 1.7	14.3 ± 1.8	14.0 ± 1.5	0.451
C-reactive protein (mg/dL)	3.4 ± 4.1	2.9 ± 3.6	4.6 ± 4.9	**0.048**
Fibrinogen (mg/dL)	405.2 ± 104.8	393.2 ± 102.0	432.3 ± 107.5	0.137
Creatinine (mg/dL)	1.0 ± 0.2	1.0 ± 0.2	1.0 ± 0.3	0.405
Troponin T (μg/L)	0.6 ± 1.4	0.7 ± 1.6	0.5 ± 0.7	0.680
HbA1c (%)	6.2 ± 1.3	6.2 ± 1.3	6.3 ± 1.3	0.409
GFR (ml/min/1.73 m^2^)	79.7 ± 19.5	80.2 ± 19.7	78.4 ± 19.2	0.968
**Concomitant medication *n* (%)**				
Aspirin	90 (100)	63 (100)	27 (100)	
Clopidogrel	2 (2)	2 (3)	0 (0)	0.360
Ticagrelor	38 (42)	26 (43)	12 (48)	0.648
Prasugrel	46 (51)	33 (54)	13 (52)	0.859
ß-blockers	79 (88)	56 (90)	23 (89)	0.793
Angiotensin converting enzyme (ACE) inhibitors/Angiotensin II receptor blockers (ARB)	78 (87)	45 (87)	24 (92)	0.482
Calcium channel-blockers	14 (16)	9 (15)	5 (19)	0.581
Proton pump Inhibitors (PPI)	70 (78)	49 (79)	21 (81)	0.854
Statins	84 (93)	59 (95)	25 (96)	0.838
Antidiabetic drugs	25 (28)	19 (31)	6 (23)	0.473
**ACS data**				
NSTE-ACS	26 (29)	17 (27)	9 (33)	0.543
STEMI	64 (71)	46 (43)	18 (67)	0.543
Number of stents per patient	1.9 ± 1.3	1.8 ± 1.2	2.1 ± 1.4	0.385
Total stent length	44.4 ± 29.1	43.7 ± 31.4	46.2 ± 23.0	0.346

*Data are reported as mean ± standard deviation (SD), n (number of patients) or percentages; miR, microRNA; CAD, coronary artery disease; HbA1c, glycated hemoglobin; GFR, glomerular filtration rate; PCI, percutaneous coronary intervention, STEMI, ST-elevation myocardial infarction; NSTE-ACS, non-ST-segment elevation acute coronary syndrome. Bold p-values indicate statistical significance.*

### Association of miR-125b With All-Cause Mortality

Based on the cut-off point of miR-125b (4.6) derived from the ROC analysis, the study population (*n* = 90) was divided into two subgroups: the low miR-125b cohort (*n* = 63), showing expression levels ≤4.6 (3.6 ± 0.7) and the high miR-125b group (*n* = 27) with plasma expression levels >4.6 (5.7 ± 1.0; *p* < 0.001).

**FIGURE 1:**
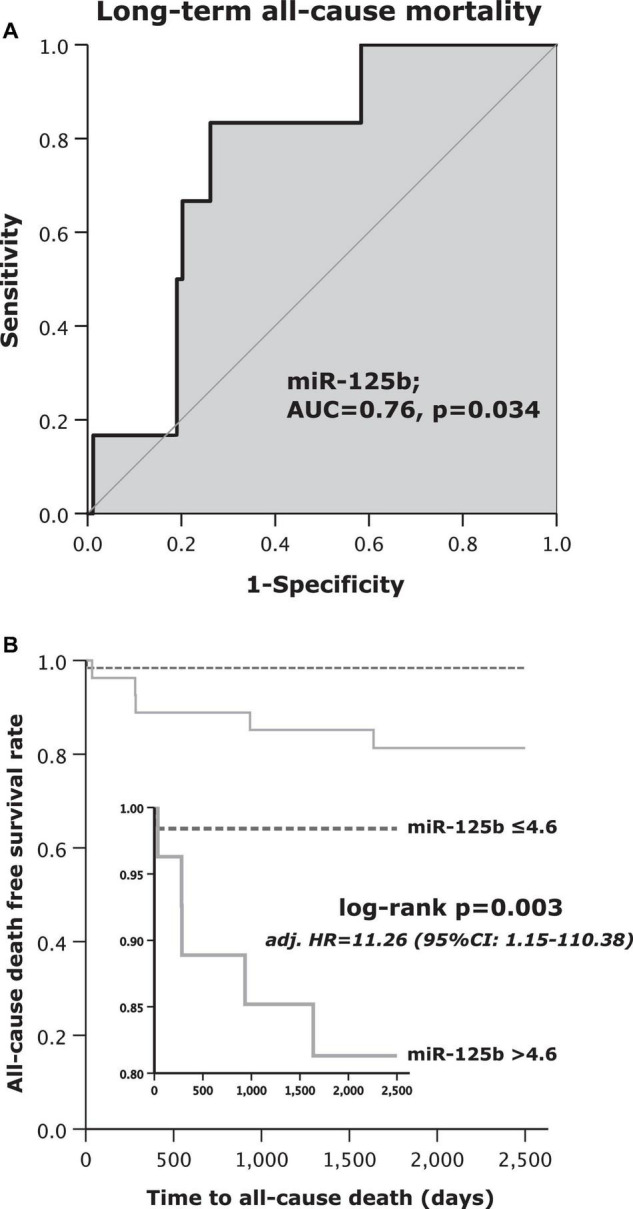
**(A)** Receiver operating curve (ROC) analysis for microRNA (miR)-125b to predict long-term all-cause mortality and **(B)** Kaplan–Meier survival analysis for long-term all-cause mortality regarding high or low values of miR-125b.

Displayed by Figure 1A, the area under the curve (AUC = c-index) was 0.76 (95% CI: 0.61–0.91; *p* = 0.034). The sensitivity of miR-125b for the prediction of long-term all-cause mortality was 83%, whereas specificity was 74%. The positive predictive value was 19%, and the negative predictive value reached 98%. Positive likelihood ratio and negative likelihood ratio were 3.2 and 0.2, respectively (Table 2).

**TABLE 2:** Statistical estimates for the prediction of long-term all-cause mortality depending on miR-125b expression levels.

*Long-term all-cause mortality n= 6 (6.7%)*									
	***Test***								
	***c-index (95% CI)***	***p-value***	***Cut-off value***	***Sensitivity, %***	***Specificity, %***	***Positive predictive value, %***	***Negative predictive value, %***	***LR+***	***LR-***
Low miR-125b vs. High miR-125b	0.76 (0.61–0.91)	**0.034**	4.6	83	74	19	98	3.2	0.2

*miR, microRNA; 95% CI = 95% confidence interval; LR +, positive likelihood ratio; LR-, negative likelihood ratio. Bold p-values indicate statistical significance.*

### Patients’ Characteristics According to Low miR-125b and High miR-125b Expression

Overall, balanced frequencies concerning risk factors, concomitant medication and data regarding the events were observed (Table 1). Within the low miR-125b subgroup, 91% were men vs. 74% in the high miR-125b (*p* = 0.043). Regarding laboratory parameters, a significant difference in CRP levels was shown between both groups: reduced CRP (2.9 ± 3.6 mg/dL) was found in patients with low miR-125b as compared to those with a high miR-125b expression (4.6 ± 4.9 md/dL; *p* = 0.048).

### Survival Analysis According to Circulating miRNAs

The primary endpoint of long-term all-cause mortality occurred in 6 out of 90 (7%) patients. Only one out of 63 patients (2%) in the low miR-125b subgroup and 5 out of 27 patients (19%) with high miR-125b levels died (Table 3). Patients with high miR-125b levels died 9.5-times more often as compared to patients with low miR-125b within the follow-up period of 7.5 years (log rank test *p* = 0.003; Figure 1B). Further, a multivariate Cox regression analysis was performed to identify independent variables for the prediction of long-term all-cause mortality (Table 4). Patients, which were assigned to the high miR-125b subgroup were at 11.26-fold increased hazard to die from all-causes in comparison to those with low miR-125b values (adjusted [adj.] hazard ratio [HR] = 11.26, 95% CI: 1.15–110.38; *p* = 0.038; Table 4). No other independent predictor for all-cause mortality was found in the performed multivariate Cox regression analysis.

**TABLE 3:** Event data.

Event	Overall *n* = 90 (100)	miR-125b ≤ 4.6 *n* = 63 (70)	miR-125b > 4.6 *n* = 27 (30)	*P*-value
*Long-term all-cause mortality*	6 (7)	1 (2)	5 (19)	**0.003**
*Long-term cardiovascular mortality*	3 (3)	1 (2)	2 (7)	0.164
*MACE (1 year)*	7 (8)	6 (10)	1 (4)	0.345
*Minimal/minor/major TIMI bleeding (1 year)*	43 (48)	29 (46)	14 (58)	0.198

*miR, microRNA; MACE, major adverse cardiac event; TIMI, Thrombolysis in Myocardial Infarction. Bold p-values indicate statistical significance.*

**TABLE 4:** Multivariate Cox regression model for prediction of long-term all-cause mortality.

Variable	HR	95% CI		*P*-value
		Lower	Upper	
*miR-125b > 4.6*	11.26	1.15	110.38	**0.038**
*Age ≥ 65 years*	3.31	0.49	22.22	0.218
*Diabetes mellitus*	0.87	0.08	9.49	0.911

*HR, hazard ratio; 95% CI, 95% confidence interval, miR, microRNA. Bold p-values indicate statistical significance.*

Receiver operating characteristic analysis showed that miR-125a and miR-223 did not have a predictive ability for long-term all-cause mortality (AUC = 0.57, 95% CI: 0.36–0.71; *p* = 0.599 and AUC = 0.47, 95% CI: 0.24–0.70; *p* = 0.783; respectively, data not shown).

### Thrombolysis for Acute Myocardial Infarction Bleeding Events Long-Term Cardiovascular Mortality and Major Adverse Cardiac Events According to miR-125b Levels

Regarding TIMI bleeding events, long-term cardiovascular mortality and MACE, no significant difference between the two subgroups was found (*p* = 0.198, *p* = 0.164 and *p* = 0.345, respectively; Table 3).

### Distribution of miR-223 and miR-125a in Regard to the Type of Acute Coronary Syndrome

**FIGURE 2:**
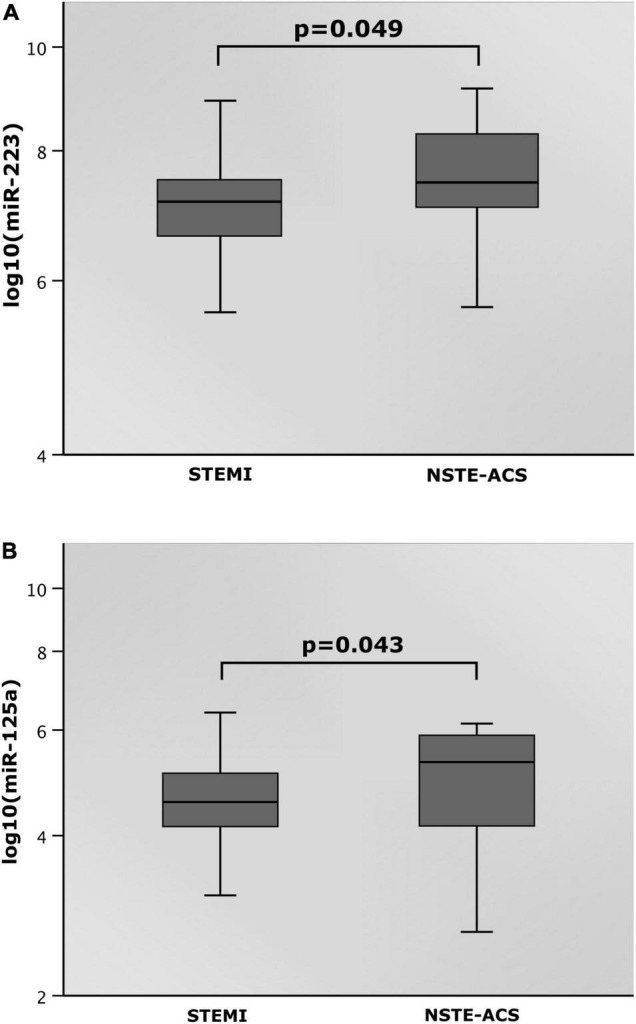
**(A)** MicroRNA (miR)-223 and **(B)** miR-125a in regard to status at hospitalization.

Both miR-223 as well as miR-125a were distributed heterogeneously between patients who presented with MVD and STEMI or NSTE-ACS, as shown by Figure 2. Median miR-223 value in the STEMI subgroup was 7.2 with an IQR of 6.6–7.5. As compared, the median miR-223 levels in patients experiencing NSTE-ACS were 4% higher (median: 7.5, IQR: 7.0–8.3; *p* = 0.049; Figure 2A). Expression of miR-125a was 13.2% higher in the NSTE-ACS subgroup (median: 5.3, IQR: 4.1–5.9) as compared to the STEMI subgroup (median: 4.6, IQR: 4.1–5.1; *p* = 0.043; Figure 2B).

### Association Between Circulating miRNAs and C-Reactive Protein Levels

**FIGURE 3:**
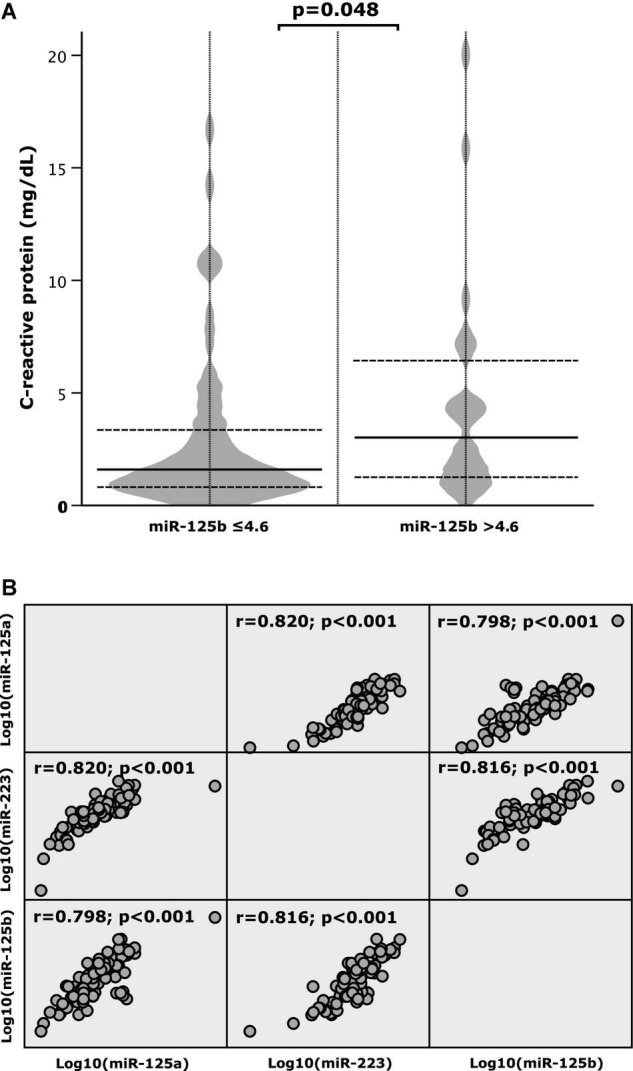
**(A)** C-reactive protein (CRP) according to high and low values of microRNA (miR)-125b and **(B)** correlation patterns between miR-125a, miR-125b, and miR-223.

There was an association between miR-125b and the inflammatory marker CRP: patients with miR-125b levels ≤4.6 also presented with lower levels of CRP (median: 1.5 mg/dL, IQR: 0.8–3.4 mg/dL), In contrast, when miR-125b exceeded the cut-off value of 4.6, CRP levels were higher (median: 3.0 mg/dL, IQR: 1.2–6.8 mg/dL; *p* = 0.048; Figure 3A). Spearman’s rho was 0.09 and not statistically significant (*p* = 0.448; data not shown).

No association between low and high levels of miR-125a (≤ 5.0; > 5.0) and CRP values was found (*p* = 0.868; data not shown).

Likewise, no correlation between high or low levels of miR-223 (≤ 7.4; > 7.4) with levels of CRP was found (*p* = 0.808; data not shown).

### Correlation Between miR-125a, miR-125b, and miR-223

Correlation patterns of miR-125a, miR-125b, and miR-223 were investigated and strong positive correlations between the respective miRNAs were determined. As depicted by Figure 3B the correlation coefficient (r) was 0.820 for miR-125a and miR-223, 0.798 for miR-125a and miR-125b and 0.816 for miR-125b and miR-223 (*p*-value < 0.001).

## Discussion

To our best knowledge, the present study is the first to demonstrate the following findings in an ACS cohort diagnosed with MVD:

i Elevation in plasma miR-125b expression in patients with STEMI or NSTE-ACS is associated with an increased risk of long-term all-cause mortality.
ii Expression of miR-125b increases direct proportionally with higher levels of CRP.
iii Expression of miR-223 and miR-125a differed between patients with STEMI vs. NSTE-ACS.

The major finding of the present study is that patients suffering from MVD had an increased odds to die from all-causes within the follow-up period of 7.5 years, when showing plasma miR-125b values >4.6. Studies already reported on the association between elevation in miR-125b and worse outcome. In 2017, it has been demonstrated that amplified expression of miR-125b predicts a poor prognosis in patients with HER2 positive breast cancer. Further, miR-125b also correlated with tumor size and TNM classification. Noteworthy, the analyses were conducted in samples collected from breast tissue, which is in contrast to our blood derived ones (41). Recently however, another study showed that increased serum miR-125b was also related to worse overall survival in patients diagnosed with non-small cell lung cancer (NSCLC) (42). Additionally, serum/plasma miR-125b was also found to be a potential marker for the prognosis prediction in sepsis patients (43) and in chronic hepatitis B patients with acute-on-chronic liver failure (44). Yet, to our knowledge this is the first study, which indicated blood derived miR-125b as a suitable predictor for long-term all-cause mortality in ACS patients suffering from MVD. One possible explanation for why an elevation in miR-125b is linked to worse outcome might be its association with inflammation (43, 45), which is also supported by our findings. The present study clearly demonstrated that upregulated miR-125b was accompanied by an increase in CRP levels in patients with MVD after ACS. Recently, similar results were obtained in severe asthma patients, where miR-125b highly correlated with high-sensitivity CRP (hsCRP) (46). Another investigation postulated a relation between increased miR-125b and elevated interleukin-6 (IL-6) in patients diagnosed with rheumatoid arthritis (47). Noteworthy, the positive correlation between IL-6 and hsCRP/CRP and its link to ACS is already largely known (48, 49).

A recent investigation indicated that miR-125b expression negatively modulates major signaling molecules of the NFκB pathway, which in turn is leading to an enhanced proinflammatory response through activation of the respective pathway (50). This would provide one possible explanation for the present findings, due to the proposed contribution of the NFκB pathway in CRP induction (51). It has also been postulated that miR-125b silencing is a feasible therapeutic option for protecting human macrophages against an infection with mycobacterium tuberculosis (52). Similarly, a recently published study reported on ameliorating non-alcoholic fatty liver disease by miR-125b inhibition (53). However, data on elevated miR-125b are inconclusive. Another study attributed positive effects to an increased miR-125b expression by diminishing sepsis-induced cardiac dysfunction and improving survival. Those effects were partly mediated by suppressing intercellular adhesion molecule 1 (ICAM-1) and vascular cell adhesion molecule-1 (VCAM-1), as well as by decreasing tumor necrosis factor-α (TNF-α) (54, 55). Noteworthy, upregulation of these molecules is associated with inflammation (56). However, this is contrary to what has been found in another trial where TNF-α and miR-125b positively correlated (43). Nonetheless, Wang and colleagues also suggested that overexpression of miR-125b mediates anti-inflammatory properties by negatively influencing the NFκB pathway (24). Since, miR-125b is obviously present in a variety of organs, one could assume that the opposing effects may depend on the miR-125b origin. However, another study on miR-125b in cardiac tissue, similar to Ma et al. (54), claimed that inhibition of miR-125b can attenuate cardiac fibrosis and may one day serves as a treatment option for this common disease (57). Based on those inconsistent findings raised in different trials, further research evaluating the exact targets and effects of miR-125b is unequivocally needed. In addition, standardization for sample generation and performance of miRNA analyses is needed, since variation of the approaches can cause different outcomes and impedes the replication of the results (58, 59).

In the investigated study cohort, circulating miR-223 and miR-125a varied between patients who were hospitalized due to STEMI or NSTE-ACS. On average, patients who suffered from NSTE-ACS have a more severe CAD as compared to those with STEMI. In our analysis, higher values of miR-223 and miR-125a were found in the NSTE-ACS population as compared to those admitted due to STEMI. Other studies have also shown that the expression of miR-223 is increased in patients with CAD as compared to controls without (60, 61) or with less severe CAD (60, 62). Li and colleagues demonstrated an upregulation of miR-223 in subjects diagnosed with acute myocardial infarction (63). However, as far as we are aware, this is the first study specifically investigating miR-223 in regard to the type of ACS. Consistently, there is also evidence that miR-125a is linked to cardiac pathologies. Patients diagnosed with HIV bear an increased risk for myocardial infarction, when showing elevated miR-125a expression (15). Moreover, it has been shown that miR-125a is also upregulated in heart failure with reduced ejection fraction (HFrEF) (64). Notwithstanding, we are the first to postulate an association between miR-125a values and the type of ACS.

However, the question remains why patients diagnosed with NSTE-ACS had an increased expression of both miR-223 and miR-125a as opposed to those who suffered from STEMI. Studies have shown that long-term outcome among patients with NSTE-ACS is in general poorer in comparison to subjects diagnosed with a STEMI (65, 66). These differences can be partly explained by the increased atherosclerotic burden in NSTE-ACS patients (67). An association between increased miR-223 plasma expression and advanced stages of atherosclerosis has been described previously (61, 68), which provides a feasible explanation for the elevated miR-223 values in those patients diagnosed with a NSTE-ACS. Atherosclerosis stimulates an increased miR-223 release of leucocytes and platelets. In this context, it is assumed that miR-223 has a protective function by mediating antiproliferative and antimigratory effects in vascular smooth muscle cells (VSMCs), which play a key role in atherosclerotic processes. Additionally, miR-223 has been shown to induce apoptosis of VSMCs, further contributing to diminished atherogenesis and neointimal formation of the vascular wall (68, 69).

Consistent to miR-223, the expression of miR-125a was also increased in patients with NSTE-ACS as compared to those with STEMI. Lipid uptake and proinflammatory processes are major targets affected by miR-125a. Oxidized low-density lipoprotein (oxLDL) has a central role in the development of atherosclerosis through promotion of inflammation and lipid deposition in the vascular wall and was demonstrated to be influenced by miR-125a (70, 71). Whereas elevated miR-125a values result in a decreased lipid uptake *via* the oxLDL-stimulated monocytes/macrophages, lipid uptake is increased, when miR-125a expression is diminished. Hence, similar to miR-223, increased plasma miR-125a expression is also considered to provide a certain protection against atherosclerosis (8)–*inter alia* also by suppressing VSMC proliferation (72).

Another possible explanation for the increased expression of miR-125a in patients with NSTE-ACS as opposed to those with STEMI might be its association to Endothelin-1 (ET-1) and atherosclerotic plaques. It has been shown that ET-1 is suppressed by miR-125a, indicating that a downregulation of the respective miRNA results in enhanced values of ET-1 (73, 74). ET-1 is an endogenous peptide, providing vast constrictive and chemoattractive properties (75, 76) and is further highly involved in the rupture of atherosclerotic plaques, which is known to be more common in lesions of STEMI than NSTE-ACS (74, 77).

In summary, we want to put emphasis on the especially high negative predictive value of low miR-125b for long-term all-cause mortality, comprising 98% in our study. That result indicates an excellent 7.5 years survival, when patients were tested with values of miR-125b lower than 4.6 at baseline. Moreover, we highlight the differences of miR-125a and miR-223 related to the ACS type, raised in our investigation, which sheds further light on the very unexplored field of miRNAs.

### Limitations

Our study points out three major limitations. Firstly, there may be bias due to the observational study design despite efforts to adjust for baseline differences by applying multivariate Cox regression analysis. Secondly, this investigation comprises a relatively small sample size of 90 participants and had a limited number of events, therefore indicating an insufficient statistical power. However, this number of patients is similar to those in other studies, that looked into miRNAs and outcome. Lastly, the required validation in an independent study cohort, which is essential for the generalizability of the results, has not been performed.

## Conclusion

The present investigation clearly demonstrates better long-term all-cause survival in patients with plasma miR-125b expression levels lower than 4.6. Further, the probability (negative predictive value of 98%) of surviving a period of 7.5 years, when low miR-125b was assessed at baseline, should be highlighted. Lastly, the current study showed the differences in expression levels of miR-125a and miR-223 depending on ACS subtype.

## Data Availability Statement

The raw data supporting the conclusions of this article will be made available by the authors, without undue reservation.

## Ethics Statement

The studies involving human participants were reviewed and approved by the Ethics Committee of the Medical University of Vienna (approval number: 1051/2012). The patients/participants provided their written informed consent to participate in this study.

## Author Contributions

GMG: writing – original draft preparation, conceptualization, methodology, investigation, visualization, and formal analysis. CE: methodology, investigation, data curation, and writing – review and editing. MP: validation, investigation, and writing – review and editing. AG and JJ-P: investigation and writing – review and editing. GG: formal analysis and writing – review and editing. BJ: validation and writing – review and editing. IL: writing – review and editing. JS-M: conceptualization, methodology, validation, investigation, writing – review and editing, supervision, and project administration. All authors contributed to the article and approved the submitted version.

## Conflict of Interest

The authors declare that the research was conducted in the absence of any commercial or financial relationships that could be construed as a potential conflict of interest.

## Publisher’s Note

All claims expressed in this article are solely those of the authors and do not necessarily represent those of their affiliated organizations, or those of the publisher, the editors and the reviewers. Any product that may be evaluated in this article, or claim that may be made by its manufacturer, is not guaranteed or endorsed by the publisher.

## References

[1] Pordzik J Eyileten-Postuła C Jakubik D Czajka P Nowak A De Rosa S MiR-126 is an independent predictor of long-term all-cause mortality in patients with type 2 diabetes mellitus. *J Clin Med.* (2021) 10:2371. 10.3390/jcm10112371 34071189 PMC8198825

[2] Soplinska A Zareba L Wicik Z Eyileten C Jakubik D Siller-Matula JM MicroRNAs as biomarkers of systemic changes in response to endurance exercise-a comprehensive review. *Diagnostics.* (2020) 10:813. 10.3390/diagnostics10100813 33066215 PMC7602033

[3] O’Brien J Hayder H Zayed Y Peng C. Overview of MicroRNA biogenesis, mechanisms of actions, and circulation. *Front Endocrinol (Lausanne).* (2018) 9:402. 10.3389/fendo.2018.00402 30123182 PMC6085463

[4] Lu TX Rothenberg ME. MicroRNA. *J Allergy Clin Immunol.* (2018) 141:1202–7. 29074454 10.1016/j.jaci.2017.08.034 PMC5889965

[5] Czajka P Fitas A Jakubik D Eyileten C Gasecka A Wicik Z MicroRNA as potential biomarkers of platelet function on antiplatelet therapy: a review. *Front Physiol.* (2021) 12:652579. 10.3389/fphys.2021.652579 33935804 PMC8081881

[6] Zareba L Fitas A Wolska M Junger E Eyileten C Wicik Z MicroRNAs and long noncoding RNAs in coronary artery disease: new and potential therapeutic targets. *Cardiol Clin.* (2020) 38:601–17. 33036721 10.1016/j.ccl.2020.07.005

[7] Jakubik D Fitas A Eyileten C Jarosz-Popek J Nowak A Czajka P MicroRNAs and long non-coding RNAs in the pathophysiological processes of diabetic cardiomyopathy: emerging biomarkers and potential therapeutics. *Cardiovasc Diabetol.* (2021) 20:55. 10.1186/s12933-021-01245-2 33639953 PMC7916283

[8] Di Mauro V Crasto S Colombo FS Di Pasquale E Catalucci D. Wnt signalling mediates miR-133a nuclear re-localization for the transcriptional control of Dnmt3b in cardiac cells. *Sci Rep.* (2019) 9:9320. 10.1038/s41598-019-45818-4 31249372 PMC6597717

[9] Yang D Wan X Dennis AT Bektik E Wang Z Costa MGS MicroRNA biophysically modulates cardiac action potential by direct binding to ion channel. *Circulation.* (2021) 143:1597–613. 10.1161/CIRCULATIONAHA.120.050098 33590773 PMC8132313

[10] Santovito D Egea V Bidzhekov K Natarelli L Mourão A Blanchet X Noncanonical inhibition of caspase-3 by a nuclear microRNA confers endothelial protection by autophagy in atherosclerosis. *Sci Transl Med.* (2020) 12:eaaz2294. 10.1126/scitranslmed.aaz2294 32493793

[11] Paul P Chakraborty A Sarkar D Langthasa M Rahman M Bari M Interplay between miRNAs and human diseases. *J Cell Physiol.* (2018) 233:2007–18. 28181241 10.1002/jcp.25854

[12] Fu G Brkić J Hayder H Peng C. MicroRNAs in human placental development and pregnancy complications. *Int J Mol Sci.* (2013) 14:5519–44. 23528856 10.3390/ijms14035519 PMC3634453

[13] Ahlin F Arfvidsson J Vargas KG Stojkovic S Huber K Wojta J. MicroRNAs as circulating biomarkers in acute coronary syndromes: a review. *Vascul Pharmacol.* (2016) 81:15–21. 27084396 10.1016/j.vph.2016.04.001

[14] Jia K Shi P Han X Chen T Tang H Wang J. Diagnostic value of miR-30d-5p and miR-125b-5p in acute myocardial infarction. *Mol Med Rep.* (2016) 14:184–94. 10.3892/mmr.2016.5246 27176713 PMC4918561

[15] Yuan N Scherzer R Tanriverdi K Martin J Rahalkar S Hsue P. MicroRNA biomarkers associated with type 1 myocardial infarction in HIV-positive individuals. *AIDS.* (2019) 33:2351–61. 10.1097/QAD.0000000000002368 31764100 PMC6905123

[16] Çakmak HA Demir M. MicroRNA and cardiovascular diseases. *Balkan Med J.* (2020) 37:60–71. 32018347 10.4274/balkanmedj.galenos.2020.2020.1.94 PMC7094181

[17] Eisen A Giugliano RP Braunwald E. Updates on acute coronary syndrome: a review. *JAMA Cardiol.* (2016) 1:718–30. 27438381 10.1001/jamacardio.2016.2049

[18] Bob-Manuel T Ifedili I Reed G Ibebuogu UN Khouzam RN. Non-ST elevation acute coronary syndromes: a comprehensive review. *Curr Probl Cardiol.* (2017) 42:266–305. 28764841 10.1016/j.cpcardiol.2017.04.006

[19] Sanchis-Gomar F Perez-Quilis C Leischik R Lucia A. Epidemiology of coronary heart disease and acute coronary syndrome. *Ann Transl Med.* (2016) 4:256. 27500157 10.21037/atm.2016.06.33 PMC4958723

[20] Belli G Presbitero P. Multivessel disease in primary percutaneous coronary intervention. *Minerva Cardioangiol.* (2012) 60:195–201. 22495168

[21] Baumann AAW Mishra A Worthley MI Nelson AJ Psaltis PJ. Management of multivessel coronary artery disease in patients with non-ST-elevation myocardial infarction: a complex path to precision medicine. *Ther Adv Chronic Dis.* (2020) 11:2040622320938527. 10.1177/2040622320938527 32655848 PMC7331770

[22] Warrington KJ Kent PD Frye RL Lymp JF Kopecky SL Goronzy JJ Rheumatoid arthritis is an independent risk factor for multi-vessel coronary artery disease: a case control study. *Arthritis Res Ther.* (2005) 7:R984–91. 10.1186/ar1775 16207339 PMC1257428

[23] Chen C-Y Lee DS Choong OK Chang S-K Hsu T Nicholson MW Cardiac-specific microRNA-125b deficiency induces perinatal death and cardiac hypertrophy. *Sci Rep.* (2021) 11:2377. 10.1038/s41598-021-81700-y 33504864 PMC7840921

[24] Wang X Ha T Zou J Ren D Liu L Zhang X MicroRNA-125b protects against myocardial ischaemia/reperfusion injury via targeting p53-mediated apoptotic signalling and TRAF6. *Cardiovasc Res.* (2014) 102:385–95. 10.1093/cvr/cvu044 24576954 PMC4030511

[25] Bayoumi AS Park KM Wang Y Teoh JP Aonuma T Tang Y A carvedilol-responsive microRNA, miR-125b-5p protects the heart from acute myocardial infarction by repressing pro-apoptotic bak1 and klf13 in cardiomyocytes. *J Mol Cell Cardiol.* (2018) 114:72–82. 10.1016/j.yjmcc.2017.11.003 29122578 PMC5800989

[26] Wang Y Tan J Wang L Pei G Cheng H Zhang Q MiR-125 family in cardiovascular and cerebrovascular diseases. *Front Cell Dev Biol.* (2021) 9:799049. 10.3389/fcell.2021.799049 34926475 PMC8674784

[27] Zhang M-W Shen Y-J Shi J Yu J-G. MiR-223-3p in cardiovascular diseases: a biomarker and potential therapeutic target. *Front Cardiovasc Med.* (2021) 7:610561. 10.3389/fcvm.2020.610561 33553260 PMC7854547

[28] Schulte C Molz S Appelbaum S Karakas M Ojeda F Lau DM miRNA-197 and miRNA-223 predict cardiovascular death in a cohort of patients with symptomatic coronary artery disease. *PLoS One.* (2015) 10:e0145930. 10.1371/journal.pone.0145930 26720041 PMC4699820

[29] Wicik Z Czajka P Eyileten C Fitas A Wolska M Jakubik D The role of miRNAs in regulation of platelet activity and related diseases - a bioinformatic analysis. *Platelets.* (2022) 1–13. 10.1080/09537104.2022.2042233 [Epub ahead of print]. 35285386

[30] Pordzik J Jakubik D Jarosz-Popek J Wicik Z Eyileten C De Rosa S Significance of circulating microRNAs in diabetes mellitus type 2 and platelet reactivity: bioinformatic analysis and review. *Cardiovasc Diabetol.* (2019) 18:113. 10.1186/s12933-019-0918-x 31470851 PMC6716825

[31] Eyileten C Wicik Z Keshwani D Aziz F Aberer F Pferschy PN Alteration of circulating platelet-related and diabetes-related microRNAs in individuals with type 2 diabetes mellitus: a stepwise hypoglycaemic clamp study. *Cardiovasc Diabetol.* (2022) 21:79. 10.1186/s12933-022-01517-5 35596173 PMC9123651

[32] De Rosa S La Bella S Canino G Siller-Matula J Eyleten C Postula M Reciprocal modulation of Linc-223 and its ligand miR-125a on the basis of platelet function level. *Eur Heart J.* (2020) 41(Suppl. 2):ehaa946.3760.

[33] Chapman AR Adamson PD Shah ASV Anand A Strachan FE Ferry AV High-sensitivity cardiac troponin and the universal definition of myocardial infarction. *Circulation.* (2020) 141:161–71. 31587565 10.1161/CIRCULATIONAHA.119.042960 PMC6970546

[34] Hicks KA Mahaffey KW Mehran R Nissen SE Wiviott SD Dunn B 2017 cardiovascular and stroke endpoint definitions for clinical trials. *Circulation.* (2018) 137:961–72. 29483172 10.1161/CIRCULATIONAHA.117.033502

[35] De Rosa R De Rosa S Leistner D Boeckel JN Keller T Fichtlscherer S Transcoronary concentration gradient of microRNA-133a and outcome in patients with coronary artery disease. *Am J Cardiol.* (2017) 120:15–24. 10.1016/j.amjcard.2017.03.264 28511772

[36] De Rosa S Eposito F Carella C Strangio A Ammirati G Sabatino J Transcoronary concentration gradients of circulating microRNAs in heart failure. *Eur J Heart Fail.* (2018) 20:1000–10. 10.1002/ejhf.1119 29314582

[37] Eyileten C Wicik Z Fitas A Marszalek M Simon JE De Rosa S Altered circulating microRNA profiles after endurance training: a cohort study of ultramarathon runners. *Front Physiol.* (2022) 12:792931. 10.3389/fphys.2021.792931 35145424 PMC8824535

[38] Uno H Cai T Pencina MJ D’Agostino RB Wei LJ. On the C-statistics for evaluating overall adequacy of risk prediction procedures with censored survival data. *Stat Med.* (2011) 30:1105–17. 10.1002/sim.4154 21484848 PMC3079915

[39] José de Carvalho Cantarelli M Castello HJ Gonçalves R Gioppato S Batista de Freitas Guimarães J Pracchia Ribeiro EK Independent predictors of multivessel coronary artery disease: results from Angiocardio registry. *Rev Bras Cardiol Invasiv.* (2015) 23:266–70.

[40] Youden WJ. Index for rating diagnostic tests. *Cancer.* (1950) 3:32–5. 15405679 10.1002/1097-0142(1950)3:1<32::aid-cncr2820030106>3.0.co;2-3

[41] Luo Y Wang X Niu W Wang H Wen Q Fan S Elevated microRNA-125b levels predict a worse prognosis in HER2-positive breast cancer patients. *Oncol Lett.* (2017) 13:867–74. 10.3892/ol.2016.5482 28356971 PMC5351301

[42] Shi GL Zhang XY Chen Y Ma S Bai WQ Yin YJ. Prognostic significance of serum miR-22, miR-125b, and miR-15b in non-small cell lung cancer patients. *Clin Lab.* (2020) 66. 10.7754/Clin.Lab.2019.191129 32538046

[43] Zhao D Li S Cui J Wang L Ma X Li Y. Plasma miR-125a and miR-125b in sepsis: correlation with disease risk, inflammation, severity, and prognosis. *J Clin Lab Anal.* (2020) 34:e23036. 32077163 10.1002/jcla.23036 PMC7031612

[44] Tao YC Wang ML Wang M Ma YJ Bai L Feng P Quantification of circulating miR-125b-5p predicts survival in chronic hepatitis B patients with acute-on-chronic liver failure. *Dig Liver Dis.* (2019) 51:412–8. 10.1016/j.dld.2018.08.030 30274791

[45] Chao C-T Yeh H-Y Yuan T-H Chiang C-K Chen H-W. MicroRNA-125b in vascular diseases: an updated systematic review of pathogenetic implications and clinical applications. *J Cell Mol Med.* (2019) 23:5884–94. 10.1111/jcmm.14535 31301111 PMC6714222

[46] Atashbasteh M Mortaz E Mahdaviani SA Jamaati H Allameh A. Expression levels of plasma exosomal miR-124, miR-125b, miR-133b, miR-130a and miR-125b-1-3p in severe asthma patients and normal individuals with emphasis on inflammatory factors. *Allergy Asthma Clin Immunol.* (2021) 17:51. 10.1186/s13223-021-00556-z 34001212 PMC8276852

[47] Zhang B Wang LS Zhou YH. Elevated microRNA-125b promotes inflammation in rheumatoid arthritis by activation of NF-κB pathway. *Biomed Pharmacother.* (2017) 93:1151–7. 10.1016/j.biopha.2017.07.042 28738524

[48] Gager GM Biesinger B Hofer F Winter MP Hengstenberg C Jilma B Interleukin-6 level is a powerful predictor of long-term cardiovascular mortality in patients with acute coronary syndrome. *Vasc Pharmacol.* (2020) 135:106806. 10.1016/j.vph.2020.106806 33035661

[49] Wang H Liu Z Shao J Lin L Jiang M Wang L Immune and inflammation in acute coronary syndrome: molecular mechanisms and therapeutic implications. *J Immunol Res.* (2020) 2020:4904217. 32908939 10.1155/2020/4904217 PMC7450309

[50] Valmiki S Ahuja V Puri N Paul J. miR-125b and miR-223 contribute to inflammation by targeting the key molecules of NFκB pathway. *Front Med (Lausanne).* (2020) 6:313. 10.3389/fmed.2019.00313 32039213 PMC6990118

[51] Agrawal A Cha-Molstad H Samols D Kushner I. Overexpressed nuclear factor-kappaB can participate in endogenous C-reactive protein induction, and enhances the effects of C/EBPbeta and signal transducer and activator of transcription-3. *Immunology.* (2003) 108:539–47. 10.1046/j.1365-2567.2003.01608.x 12667216 PMC1782914

[52] Liu G Wan Q Li J Hu X Gu X Xu S. Silencing miR-125b-5p attenuates inflammatory response and apoptosis inhibition in mycobacterium tuberculosis-infected human macrophages by targeting DNA damage-regulated autophagy modulator 2 (DRAM2). *Cell Cycle.* (2020) 19:3182–94. 10.1080/15384101.2020.1838792 33121314 PMC7714508

[53] Zhang Q Yu K Cao Y Luo Y Liu Y Zhao C. miR-125b promotes the NF-κB-mediated inflammatory response in NAFLD via directly targeting TNFAIP3. *Life Sci.* (2021) 270:119071. 10.1016/j.lfs.2021.119071 33515562

[54] Ma H Wang X Ha T Gao M Liu L Wang R MicroRNA-125b prevents cardiac dysfunction in polymicrobial sepsis by targeting TRAF6-mediated nuclear factor κB activation and p53-mediated apoptotic signaling. *J Infect Dis.* (2016) 214:1773–83. 10.1093/infdis/jiw449 27683819 PMC5144735

[55] Zelová H Hošek J. TNF-α signalling and inflammation: interactions between old acquaintances. *Inflamm Res.* (2013) 62:641–51. 10.1007/s00011-013-0633-0 23685857

[56] Gager GM von Lewinski D Sourij H Jilma B Eyileten C Filipiak K Effects of SGLT2 inhibitors on Ion homeostasis and oxidative stress associated mechanisms in heart failure. *Biomed Pharmacother.* (2021) 143:112169. 10.1016/j.biopha.2021.112169 34560555

[57] Nagpal V Rai R Place AT Murphy SB Verma SK Ghosh AK MiR-125b is critical for fibroblast-to-myofibroblast transition and cardiac fibrosis. *Circulation.* (2016) 133:291–301. 26585673 10.1161/CIRCULATIONAHA.115.018174 PMC5446084

[58] Santovito D Weber C. Zooming in on microRNAs for refining cardiovascular risk prediction in secondary prevention. *Eur Heart J.* (2016) 38:524–8. 10.1093/eurheartj/ehw259 27371715

[59] Zampetaki A Mayr M. Analytical challenges and technical limitations in assessing circulating miRNAs. *Thromb Haemost.* (2012) 108:592–8. 22627831 10.1160/TH12-02-0097

[60] Saadatian Z Nariman-Saleh-Fam Z Bastami M Mansoori Y Khaheshi I Parsa SA Dysregulated expression of STAT1, miR-150, and miR-223 in peripheral blood mononuclear cells of coronary artery disease patients with significant or insignificant stenosis. *J Cell Biochem.* (2019) 120:19810–24. 31318097 10.1002/jcb.29286

[61] Guo JF Zhang Y Zheng QX Zhang Y Zhou HH Cui LM. Association between elevated plasma microRNA-223 content and severity of coronary heart disease. *Scand J Clin Lab Invest.* (2018) 78:373–8. 10.1080/00365513.2018.1480059 29888618

[62] Singh S de Ronde MWJ Kok MGM Beijk MA De Winter RJ van der Wal AC MiR-223-3p and miR-122-5p as circulating biomarkers for plaque instability. *Open Heart.* (2020) 7:e001223. 10.1136/openhrt-2019-001223 32487772 PMC7269547

[63] Li C Fang Z Jiang T Zhang Q Liu C Zhang C Serum microRNAs profile from genome-wide serves as a fingerprint for diagnosis of acute myocardial infarction and angina pectoris. *BMC Med Genomics.* (2013) 6:16. 10.1186/1755-8794-6-16 23641832 PMC3655858

[64] Wong LL Armugam A Sepramaniam S Karolina DS Lim KY Lim JY Circulating microRNAs in heart failure with reduced and preserved left ventricular ejection fraction. *Eur J Heart Fail.* (2015) 17 393–404. 25619197 10.1002/ejhf.223

[65] Chan MY Sun JL Newby LK Shaw LK Lin M Peterson Long-term mortality of patients undergoing cardiac catheterization for ST-elevation and non-ST-elevation myocardial infarction. *Circulation.* (2009) 119:3110–7. 19506116 10.1161/CIRCULATIONAHA.108.799981

[66] Ishihara M Nakao K Ozaki Y Kimura K Ako J Noguchi T Long-term outcomes of non-ST-elevation myocardial infarction without creatine kinase elevation- the J-MINUET study. *Circ J.* (2017) 81:958–65. 10.1253/circj.CJ-17-0033 28320999

[67] Tanaka T Miki K Akahori H Imanaka T Yoshihara N Kimura T Comparison of coronary atherosclerotic disease burden between ST-elevation myocardial infarction and non-ST-elevation myocardial infarction: non-culprit Gensini score and non-culprit SYNTAX score. *Clin Cardiol.* (2021) 44:238–43. 10.1002/clc.23534 33368316 PMC7852165

[68] Shan Z Qin S Li W Wu W Yang J Chu M An endocrine genetic signal between blood cells and vascular smooth muscle cells: role of microRNA-223 in smooth muscle function and Atherogenesis. *J Am Coll Cardiol.* (2015) 65:2526–37. 10.1016/j.jacc.2015.03.570 26065992 PMC4498487

[69] Chistiakov DA Orekhov AN Bobryshev YV. Vascular smooth muscle cell in atherosclerosis. *Acta Physiol (Oxf).* (2015) 214:33–50. 25677529 10.1111/apha.12466

[70] Trpkovic A Resanovic I Stanimirovic J Radak D Mousa SA Cenic-Milosevic D Oxidized low-density lipoprotein as a biomarker of cardiovascular diseases. *Crit Rev Clin Lab Sci.* (2015) 52:70–85. 25537066 10.3109/10408363.2014.992063

[71] Chen T Huang Z Wang L Wang Y Wu F Meng S MicroRNA-125a-5p partly regulates the inflammatory response, lipid uptake, and ORP9 expression in oxLDL-stimulated monocyte/macrophages. *Cardiovasc Res.* (2009) 83:131–9. 10.1093/cvr/cvp121 19377067

[72] Ye D Lou GH Li AC Dong FQ Chen GP Xu WW MicroRNA-125a-mediated regulation of the mevalonate signaling pathway contributes to high glucose-induced proliferation and migration of vascular smooth muscle cells. *Mol Med Rep.* (2020) 22:165–74. 10.3892/mmr.2020.11077 32319638 PMC7248521

[73] Li D Yang P Xiong Q Song X Yang X Liu L MicroRNA-125a/b-5p inhibits endothelin-1 expression in vascular endothelial cells. *J Hypertens.* (2010) 28:1646–54. 10.1097/HJH.0b013e32833a4922 20531225

[74] Hao L Wang X-G Cheng J-D You S-Z Ma S-H Zhong X The up-regulation of endothelin-1 and down-regulation of miRNA-125a-5p, −155, and –199a/b-3p in human atherosclerotic coronary artery. *Cardiovasc Pathol.* (2014) 23:217–23. 10.1016/j.carpath.2014.03.009 24877885

[75] Houde M Desbiens L D’Orléans-Juste P. Endothelin-1: biosynthesis, signaling and vasoreactivity. *Adv Pharmacol.* (2016) 77:143–75. 27451097 10.1016/bs.apha.2016.05.002

[76] Chen P Shibata M Zidovetzki R Fisher M Zlokovic BV Hofman FM. Endothelin-1 and monocyte chemoattractant protein-1 modulation in ischemia and human brain-derived endothelial cell cultures. *J Neuroimmunol.* (2001) 116:62–73. 10.1016/s0165-5728(01)00280-6 11311331

[77] Dong L Mintz GS Witzenbichler B Metzger DC Rinaldi MJ Duffy PL Comparison of plaque characteristics in narrowings with ST-elevation myocardial infarction (STEMI), non-STEMI/unstable angina pectoris and stable coronary artery disease (from the ADAPT-DES IVUS substudy). *Am J Cardiol.* (2015) 115:860–6. 10.1016/j.amjcard.2015.01.008 25661569

